# Obesity and outcome of post-menopausal women receiving adjuvant letrozole for breast cancer

**DOI:** 10.3332/ecancer.2018.821

**Published:** 2018-03-26

**Authors:** Jamal Zekri, Kamel Farag, Ahmed Allithy

**Affiliations:** 1 College of Medicine, Al-Faisal University, Riyadh 11533, Saudi Arabia; 2 King Faisal Specialist Hospital & Research Centre, Jeddah 21499, Saudi Arabia; 3 Mansoura University, Mansoura, Egypt

**Keywords:** breast cancer, obesity, post-menopausal, adjuvant, letrozole

## Abstract

**Introduction:**

Aromatase enzyme activity is predominant in adipose tissue. This has led to speculation that aromatase activity is elevated in obese women and subsequently decreased the clinical activity of adjuvant aromatase inhibitors (AIs) in women with estrogen receptor positive (ER+) breast cancer (BC). We investigated the effect of obesity on the outcome of this population.

**Patients and methods:**

Records of 320 consecutive post-menopausal (PM) women with ER+ BC starting single agent adjuvant letrozole between years 2005 and 2014 were retrospectively reviewed. Tumour and patients characteristic including body mass index (BMI) on the day of starting letrozole were extracted. Endpoints of main interest were: (1) Frequency of obesity; (2) relapse-free survival (RFS) in nonobese (G1; BMI < 30) and obese (G2; BMI ≥ 30) patients.

**Results:**

Obesity (BMI: 30–34.99) and morbid obesity (BMI ≥ 35) were present in 105/320 (32.8%) and 115/320 (35.9%) women, respectively. Median follow-up of patients was 49 months; RFS at 5 years (G1: 69% versus G2: 78%) and at 8 years (G1: 69% versus G2: 71%). Median RFS is not reached in both groups (Log rank; *P* = 0.097). There was no correlation between BMI and RFS (correlation coefficient *r* = 0.075; *P* = 0.174).

**Conclusion:**

In this cohort, more than two-thirds of PM women starting adjuvant AIs are obese. Obesity did not adversely affect the outcome of women on adjuvant letrozole.

## Introduction

Breast cancer (BC) is the most commonly diagnosed cancer and is a leading cause of mortality among women worldwide [1]. In patients with estrogen receptor positive (ER+) tumours, the outcome can be improved after surgery by depriving microscopic disease from estrogen through inhibiting ERs or estrogen production. Tamoxifen, a selective ER inhibitor, improves relapse-free survival (RFS) and overall survival (OS) in these patients [2]. Aromatisation of androgens accounts for the peripheral formation of significant proportion of estrogen in post-menopausal (PM) women. For this reason, aromatase inhibitors (AIs) were extensively investigated in the adjuvant setting. Results of randomised clinical trials confirmed the superiority of adjuvant AIs when compared with tamoxifen in PM women [3]. Thus, AIs (letrozole, anastrazole and exemestane) have become the standard adjuvant hormonal treatment in PM women with ER+BC.

Aromatase enzyme activity is predominant in adipose tissue in PM women [4]. This has led to speculation that overweight and obesity are associated with elevated aromatase activity and subsequently decreased the clinical activity of AIs in these women compared with nonobese women [5, 6].

The prevalence of obesity is increasing in many parts of the world [7]. Multinational research shows increasing levels of obesity that are most pronounced in the Middle East including Saudi Arabia [8]. Limited local studies in Saudi Arabia confirmed obesity [body mass index (BMI) ≥ 30] in 40% of women from the general population and in more than half of those with BC [9, 10].

Results of the studies investigating the effect of BMI on clinical outcome of women with early BC on adjuvant AIs are not conclusive [11]. Certainly, there are no such studies in women from Saudi Arabia. We aim to study the frequency of obesity and the relation between BMI and BC outcome in PM women on adjuvant letrozole.

## Patients and methods

The electronic and paper records of all consecutive patients with PM women with ER+ BC starting adjuvant letrozole between January 2005 and December 2014 in one hospital were retrospectively reviewed. Patients who received sequential/switching therapy (AI and tamoxifen) and those lost to follow-up within 6 months of starting treatment were excluded. All patients were assessed and managed by a specialist BC multi-disciplinary team in a routine clinical setting according to local guidelines. Letrozole was started at a daily dose of 2.5 mg with intention to continue for 5 years or until BC relapse.

Individual patients’ data were extracted including: BMI on the day of starting letrozole, patients’ and tumours’ characteristics and disease outcome. The co-primary endpoints are (1) frequency of obesity in this population and (2) comparing RFS in nonobese (Group 1: G1; BMI <30 kg/m^2^) and obese (Group 2: G2; BMI ≥ 30 kg/m^2^) patients. Additional outcome analysis was performed to ascertain that the studied cohort reflects the known behaviour of early ER+BC in general. This was achieved by analysing RFS according to standard clinical prognostic factors (tumour size and grade and lymph node involvement). SPSS.20 software was used for statistical analysis including Kaplan–Meier and log-rank tests for survival outcome. Chi-squared test (χ^2^) was employed to evaluate categorical values and *T*-test for continuous variables differences between G1 and G2. Spearman’s test was used to investigate the correlation between BMI and RFS as continuous variables. All data collection and analysis were carried out by three medical oncologists who are the authors of this report. Institutional review board approval was granted for this study.

## Results

Three hundred and fifty-four PM women with ER+ BC starting adjuvant letrozole were identified. Thirty-four (9.6%) received sequential/switching hormonal therapy or had short follow-up and thus 320 patients met the full inclusion criteria and are the subject of this study. For the whole eligible cohort, median age is 58 (45–95) years [mean: 60.3], 10.9% of tumours over-express Her2 receptor (immunohistochemistry+++ and/or fluorescent *in situ* hybridization) and 30.9% of tumours did not involve regional lymph nodes (pathological stage N0). Table 1 depicts detailed characteristics for the whole cohort and for patients in each BMI group. There was no statistically significant difference between both groups in age (*T*-test; *P* = 0.117) and type of surgery, histology, grade, Her2 receptors, which involved lymph nodes and tumour size (Chi-square test; *P* = 0.810, 0.327, 0.494, 0.116, 0.991 and 0.161, respectively).

Mean BMI of the whole cohort was 33.3 (18.2–58.2) [median: 32.8]. BMI of 100 (31.25%) patients was in the nonobese range (G1), while that of 220 (68.8%) was in the obese range (G2). Class I (BMI: 30–34.99) and class II/III (BMI ≥ 35) obesity were present in 105 and 115 patients, respectively, in G2.

**Figure 1.**
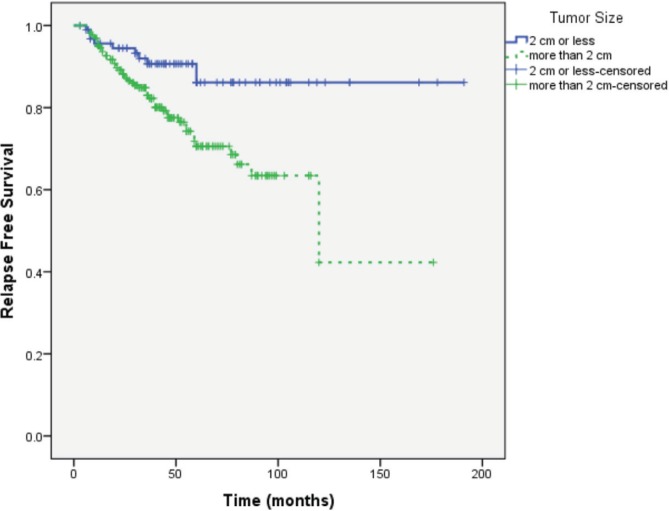
RFS according to primary tumour size.

**Figure 2.**
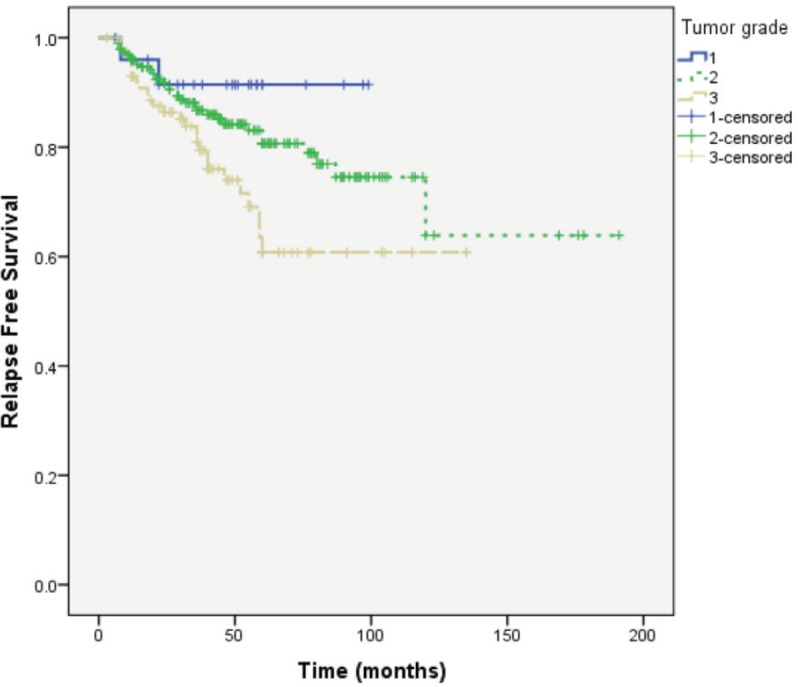
RFS according to primary tumour grade.

**Figure 3.**
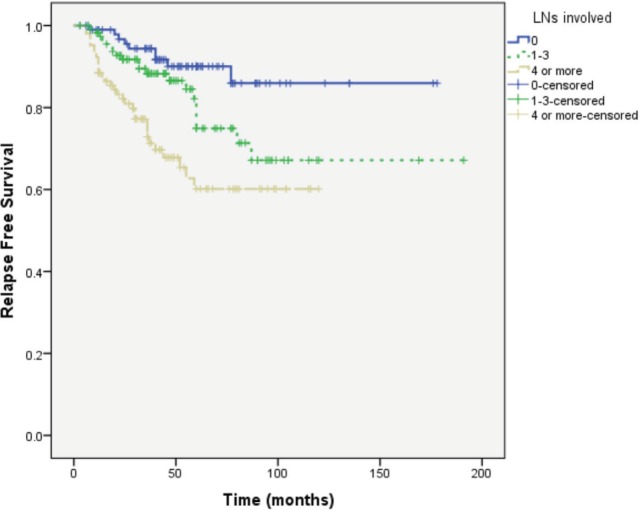
RFS according to the number of involved axillary lymph nodes.

Median follow-up of patients was 49 months. RFS was longer in patients with primary tumour size ≤ 2 cm, lower tumour grade and no or lower nodal involvement compared to those with tumour size >2 cm (*P* = 0.004), higher tumour grade (*P* = 0.037) and higher nodal involvement (*P* < 0.0001), respectively (Figures 1–3).

**Figure 4.**
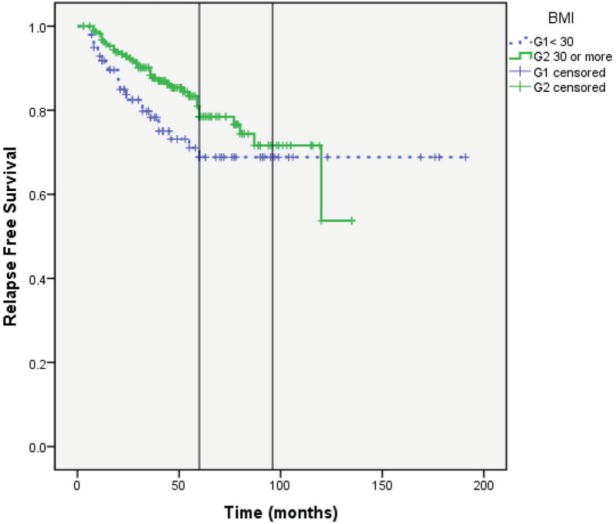
RFS in Group 1: BMI < 30 and Group 2: BMI ≥ 30.

RFS at 5 years (G1: 69% versus G2: 78%) and at 8 years (G1: 69% versus G2: 71%). Median RFS was not reached in both groups (Log Rank; *P* = 0.097) (Figure 4).

**Figure 5.**
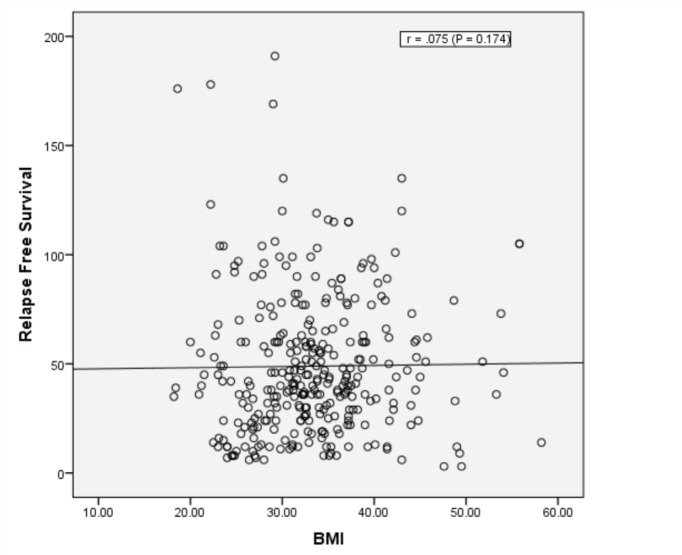
Pearson correlation between BMI and RFS.

There was no correlation between BMI as continuous variables and RFS (Correlation Coefficient *r* = 0.075; *P* = 0.174) (Figure 5).

## Discussion

There is now substantial evidence that obesity is a risk factor for the development of BC in PM women [12]. While premenopausal women mainly synthesise estrogens in the ovaries, after menopause, ovarian biosynthesis is largely replaced by peripheral sites synthesis with the adipose tissue being the main source. The primary mediator of PM estrogen biosynthesis is the aromatase enzyme [13]. In PM women, androgens produced by the adrenal cortex and the PM ovary are converted into estrogens by aromatase [4, 14, 15]. Elevated serum estrogen levels and enhanced local production of estrogen in breast tissue explain how increased body weight promotes BC development in these women.

The median BMI of our 320 eligible patients was 32.8 (range: 18.2–58.2) which is similar to that of the 34 ineligible/excluded patients (33.7, range: 20–55.8) indicating lack of selection bias. The results indicate that two-thirds (68.75%) of PM women with ER+ BC from Saudi Arabia are obese which is higher than what was reported (40%) in the general Saudi female population [9]. This is in line with the findings of the Women’s Health Initiative clinical trials confirming the association of obesity had an increased risk of invasive BC risk in 67,142 PM women [16].

AIs are the standard adjuvant hormonal treatments in PM women with ER+BC [3]. The excess aromatase activity in obese women raised speculations that standard doses of AIs may not be as effective as they are in nonobese women. Serum estrogen levels are higher in obese than in that nonobese PM women. Lønning *et al* [17] confirmed the parallel relation between BMI and serum estrogen before and during AI therapy. However, there was no relation between BMI and aromatase activity. Results of studies investigating the relation between BMI and clinical efficacy of AIs have been contradicting [18, 19].

Our results show that there is no detrimental effect of higher BMI on BC specific outcome. In fact, patients with higher BMI had numerically superior RFS during the first 5 years. This difference became less apparent at 8 years (Figure 4). Benefit from adjuvant AIs and tamoxifen can increase overtime many years after stopping the treatment [20, 21]. For this reason, it will be interesting to observe the outcome of our patients after a longer follow-up (e.g., after 10–15 years).

The lack of detrimental effect of obesity was consistent when the cohort was divided into a dichotomous manner according to WHO classification of obesity and when BMI was tested as a continuous variable (Figures 4 and 5).

In view of these results, it was imperative to ensure the validity of the collected information. We analysed the outcome of our patients according to established prognostic factors (tumour size and grade and number of involved lymph nodes). This analysis showed that the outcome of patients in our cohort is parallel to what is universally recognised with longer RFS in patients with smaller and lower grade tumours and in those with and no or lower nodal involvement (Figures 1–3).

ATAC was a large randomised phase-III trial that compared adjuvant anastrazole to tamoxifen in women with BC. After a median follow-up of 100 months, women receiving anastrozole had lower recurrence rate than those on tamoxifen (HR, 0.73; 95% CI, 0.63–0.83; *P* < 0.001).

However, the benefit of anastrozole compared with tamoxifen was greater in thinner women. For women with BMI < 23, the adjusted HR comparing anastrozole with tamoxifen was 0.64 (95% CI, 0.45–0.91), whereas for women with BMI > 30 kg/m^2^ the adjusted HR was 0.84 (95% CI, 0.61–1.14). This analysis ignores a large set of patients (53%) with BMI between 23 and 30. However, investigators divided the patients into what seems to be arbitrary six BMI groups. Increasing BMI in these six groups was associated with increasing risk of recurrence (x^2^ P for trend = 0.001) in women receiving anastrazole. In their conclusion, the authors suggested that the relative efficacy of anastrozole compared to tamoxifen is greater in thin PM women and higher doses or more complete inhibitors might be more effective in overweight women, but this requires independent confirmation [19].

The BIG 1-98 was a large randomised phase-III trial that compared adjuvant letrozole to tamoxifen. Information on BMI at randomisation was available for 4,760 patients. After a median follow-up of 8.7 years, the RFS was not statistically different between women with normal weight, overweight and obese BMI (Gray’s *P* = 0.81). The authors concluded that there is no evidence that the benefit of letrozole over tamoxifen differed according to patients’ BMI [22].

A smaller retrospective study of 501 patients who received adjuvant anastrazole (42.5%) or letrozole (57.5%) investigated the relation of BMI to the treatment outcome. The 3-year RFS was 77.6% and 85.5% in normal weight and overweight/obese patients, respectively (*P* = 0.08) [23].

It is difficult to explain the contradictory findings of these two large trials (ATAC and BIG 1-98) and other smaller studies (including ours). Several studies have reported that estrogen suppression is more complete with letrozole than that with anastrozole [24, 25]. One possible explanation is that letrozole is sufficiently active to overcome any incomplete suppression seen with anastrozole. This explanation is reassuring because it indicates that the standard dose of letrozole is sufficient to inhibit the excess amount of estrogens that obese women produce from peripheral aromatisation. Saying this, it is important to note that there is no firm evidence to indicate any difference in the clinical activity between anastrazole and letrozole. An open-label phase-III b/IV study randomised 713 patients with advanced BC had progressed on antioestrogen therapy to anastrazole or letrozole. The primary efficacy endpoint was time to progression (TTP). There was no difference between the treatment arms in TTP, clinical benefit, median duration of response, duration of clinical benefit, time to treatment failure or OS [26]. Other studies also reported equivalent efficacy of both AIs [27, 28]. The Letrozole (Femara) Versus Anastrozole Clinical Evaluation Study, a randomised phase-III b trial, was designed to compare the efficacy of adjuvant letrozole to anastrozole in 4,136 patients with node positive BC. With 709 of the protocol-planned 959 events, 5-year estimated RFS rate was 84.9% for letrozole versus 82.9% for anastrazole (HR = 0.93 [95% CI: 0.80–1.07]; *p* = 0.3150). Five-year estimated OS rate was 89.9% for letrozole versus 89.2% for anastrazole (HR = 0.98 [95% CI: 0.82–1.17]; *p* = 0.7916). Authors concluded that both AIs have equivalent efficacy [29].

Suboptimal adherence to treatment is considered to be an important clinical issue. Adherence of patients with BC to oral hormonal therapy was analysed in a systematic review of 24 studies. The authors reported a wide range of adherence and discontinuation rates, ranging from 45 to 95.7% and 12 to 73 %, respectively [30]. It was not possible to ascertain the rate of adherence in our study, which may be considered a limitation. However, adherence would have unlikely affected our results as previous literature showed that adherence to adjuvant hormonal therapy is not associated with BC outcomes [31]. Generally, financial restrictions can lead to treatment noncompliance. However, this restriction did not apply to our patients as they were treated in a public hospital providing free health care and medication.

The retrospective design and the relatively small sample size from a single institution can be considered as limitations in our study. On the other hand, the strengths are: (a) It represents all eligible sequential patients in real-life practice; (b) the validity of the collected information was scrutinised and found to be representative of behaviour of BC; and (c) median follow-up was relatively long at 49 months. However, longer follow-up may be indicated as discussed above.

## Conclusion

In this study population, obesity is common in PM women starting adjuvant AIs for BC. Obesity did not adversely affect the benefit from adjuvant single agent letrozole. This issue may warrant further investigation in the future if the currently ongoing trials support combining adjuvant AIs with other biological agents such as mTOR and CDK4/6 inhibitors.

## Conflicts of interest

The authors declare that there is no conflict of interest that could be perceived as prejudicing the impartiality of this research.

## Figures and Tables

**Table 1.** Patients’ and tumours’ characteristics.

	Overall cohort (*n* = 320)	BMI < 30 (*n* = 100)	BMI ≥ 30 (*n* = 220)
Age in years Mean Median	60.3 (45–95) 58	59.2 (45–92) 57	60.8 (45–95) 59
Breast surgery Conservative Mastectomy	163 (50.9%) 157 (49.1%)	52 (52%) 48 (48%)	111 (50.5%) 109 (49.5%)
Histology Invasive ductal carcinoma Invasive lobular carcinoma Mixed	290 (90.6%) 23 (7.2%) 7 (2.2%)	89 (89%) 7 (7%) 4 (4%)	201 (91.4%) 23 (7.2%) 7 (2.2%)
Grade I II III	26 (8.1%) 192 (60%) 130 (32%)	10 (10%) 62 (62%) 28 (28%)	16 (7.3%) 130 (59.1%) 74 (33.6%)
Her 2 Over-expressed Not over-expressed	35 (10.9%) 285 (89.1%)	15 (15%) 85 (85%)	20 (9.1%) 200 (90.9)
Involved LNs 0 1–3 ≥4	99 (30.9%) 117 (36.6%) 104 (32.5%)	31 (31%) 37 (37%) 32 (32%)	68 (30.9%) 80 (36.4%) 72 (32.7%)
Tumour size ≤2 cm >2 cm	95 (29.7%) 225 (70.3%)	35 (35%) 65 (65%)	60 (27.3%) 160 (72.7%)

## References

[1] Torre LA, Bray F, Siegel RL (2015). Global cancer statistics, 2012. CA Cancer J Clin.

[2] Davies C, Godwin J, Gray R (2011). Relevance of breast cancer hormone receptors and other factors to the efficacy of adjuvant tamoxifen: patient-level meta-analysis of randomised trials. Lancet.

[3] Dowsett M, Forbes JF, Bradley R (2015). Aromatase inhibitors versus tamoxifen in early breast cancer: patient-level meta-analysis of the randomised trials. Lancet.

[4] Bulun SE, Chen D, Moy I (2012). Aromatase, breast cancer and obesity: a complex interaction. Trends Endocrinol Metab.

[5] Schech A, Yu S, Goloubeva O (2015). A nude mouse model of obesity to study the mechanisms of resistance to aromatase inhibitors. Endocr Relat Cancer.

[6] Azrad M, Demark-Wahnefried W (2014). The association between adiposity and breast cancer recurrence and survival: a review of the recent literature. Curr Nutr Rep.

[7] Seidell JC, Halberstadt J (2015). The global burden of obesity and the challenges of prevention. Ann Nutr Metab.

[8] Arnold M, Leitzmann M, Freisling H (2016). Obesity and cancer: an update of the global impact. Cancer Epidemiol.

[9] Garawi F, Ploubidis GB, Devries K (2015). Do routinely measured risk factors for obesity explain the sex gap in its prevalence? Observations from Saudi Arabia. BMC Public Health.

[10] Al Saeed EF, Ghabbban AJ, Tunio MA (2015). Impact of BMI on locoregional control among Saudi patients with breast cancer after breast conserving surgery and modified radical mastectomy. Gulf J Oncolog.

[11] Goodwin PJ, Pritchard KI (2010). Obesity and hormone therapy in breast cancer: an unfinished puzzle. J Clin Oncol.

[12] Cleary MP, Grossmann ME (2009). Minireview: obesity and breast cancer: the estrogen connection. Endocrinology.

[13] Simpson ER, Ackerman GE, Smith ME (1981). Estrogen formation in stromal cells of adipose tissue of women: induction by glucocorticosteroids. Proc Natl Acad Sci U S A.

[14] Baird DT, Uno A, Melby JC (1969). Adrenal secretion of androgens and oestrogens. J Endocrinol.

[15] Judd HL, Judd GE, Lucas WE (1974). Endocrine function of the postmenopausal ovary: concentration of androgens and estrogens in ovarian and peripheral vein blood. J Clin Endocrinol Metab.

[16] Neuhouser ML, Aragaki AK, Prentice RL (2015). Overweight, obesity, and postmenopausal invasive breast cancer risk. JAMA Oncol.

[17] Lønning PE, Haynes BP, Dowsett M (2014). Relationship of body mass index with aromatisation and plasma and tissue oestrogen levels in postmenopausal breast cancer patients treated with aromatase inhibitors. Eur J Cancer.

[18] Michaud LB, Buzdar AU, Rubin S (2002). The efficacy of anastrozole is not dependent upon body mass index (BMI) in postmenopausal women with advanced breast cancer (BC). Proc Am Soc Clin Oncol.

[19] Sestak I, Distler W, Forbes JF (2010). Effect of body mass index on recurrences in tamoxifen and anastrozole treated women: an exploratory analysis from the ATAC trial. J Clin Oncol.

[20] Tannock IF (2011). 10-year analysis of the ATAC trial: wrong conclusion?. Lancet Oncol.

[21] Davies C, Pan H, Godwin J (2013). Long-term effects of continuing adjuvant tamoxifen to 10 years versus stopping at 5 years after diagnosis of oestrogen receptor-positive breast cancer: ATLAS, a randomised trial. Lancet.

[22] Ewertz M, Gray KP, Regan MM (2012). Obesity and risk of recurrence or death after adjuvant endocrine therapy with letrozole or tamoxifen in the breast international group 1-98 trial. J Clin Oncol.

[23] Sendur MAN, Aksoy S, Zengin N (2012). Efficacy of adjuvant aromatase inhibitor in hormone receptor-positive postmenopausal breast cancer patients according to the body mass index. Br J Cancer.

[24] Geisler J, Helle H, Ekse D (2008). Letrozole is superior to anastrozole in suppressing breast cancer tissue and plasma estrogen levels. Clin Cancer Res.

[25] Dixon JM, Renshaw L, Young O (2008). Letrozole suppresses plasma estradiol and estrone sulphate more completely than anastrozole in postmenopausal women with breast cancer. J Clin Oncol.

[26] Rose C, Vtoraya O, Pluzanska A (2003). An open randomised trial of second-line endocrine therapy in advanced breast cancer. Comparison of the aromatase inhibitors letrozole and anastrozole. Eur J Cancer.

[27] Ellis MJ, Suman VJ, Hoog J (2011). Randomized phase II neoadjuvant comparison between letrozole, anastrozole, and exemestane for postmenopausal women with estrogen receptor-rich stage 2 to 3 breast cancer: clinical and biomarker outcomes and predictive value of the baseline PAM50-based int. J Clin Oncol.

[28] Murray J, Young OE, Renshaw L (2009). A randomised study of the effects of letrozole and anastrozole on oestrogen receptor positive breast cancers in postmenopausal women. Breast Cancer Res Treat.

[29] Smith I, Yardley D, Burris H (2017). Comparative efficacy and safety of adjuvant letrozole versus anastrozole in postmenopausal patients with hormone receptor-positive, node-positive early breast cancer: final results of the randomized phase III femara versus anastrozole clinical evaluation. J Clin Oncol.

[30] Ayres LR, Baldoni A de O, Borges AP de S (2014). Adherence and discontinuation of oral hormonal therapy in patients with hormone receptor positive breast cancer. Int J Clin Pharm.

[31] Weaver KE, Camacho F, Hwang W (2013). Adherence to adjuvant hormonal therapy and its relationship to breast cancer recurrence and survival among low-income women. Am J Clin Oncol.

